# *In Vivo* Bioimaging and Biodistribution of DiR-Labeled Solid SNEDDS of Black Sticky Rice Extract Using Neusilin® US2 as Solid Carrier

**DOI:** 10.7150/ntno.127165

**Published:** 2026-04-16

**Authors:** Anggun Hari Kusumawati, Elfahmi Elfahmi, Afrillia Nuryanti Garmana, Rachmat Mauludin

**Affiliations:** 1 Pharmaceutics Department, School of Pharmacy, Institut Teknologi Bandung, Jalan Ganesha No. 10, Bandung 40132, West Java, Indonesia.; 2 Department of Pharmacognosy and Phytochemistry, School of Pharmacy, Institut Teknologi Bandung, Jalan Ganesha No. 10, Bandung 40132, West Java, Indonesia.; 3 Pharmacology-Clinical Pharmacy Department, School of Pharmacy, Institut Teknologi Bandung, Jalan Ganesha No. 10, Bandung 40132, West Java, Indonesia.; 4 Department of Pharmaceutical Technology, Faculty of Pharmacy, Universitas Buana Perjuangan Karawang, Jalan H. S. Ronggowaluyo, Karawang 41361, West Java, Indonesia.; 5 Center of Excellence for Innovative Cosmeceuticals and Natural Medicines for Degenerative Disease, Center for Pharma Valorisation, School of Pharmacy, Bandung Insitute of Technology, Bandung, Indonesia.

**Keywords:** SNEDDS, Black Sticky Rice Extract, IVIS imaging, biodistribution, radiant efficiency, hepatoprotective, polyphenols, anthocyanins

## Abstract

Black Sticky Rice Extract (BSRE; *Oryza sativa* var. *glutinosa* Blanco) is rich in anthocyanins and flavonoids with well-documented antioxidant and hepatoprotective activities. Nevertheless, its clinical translation is hindered by poor aqueous solubility and limited stability under physiological conditions, resulting in low oral bioavailability. To overcome these limitations, a Self-Nanoemulsifying Drug Delivery System (SNEDDS) was developed to enhance BSRE solubilization, stability, and hepatic targeting efficiency. A 1,1′-dioctadecyl-3,3,3′,3′-tetramethylindotricarbocyanine iodide (DiR)-labeled SNEDDS was formulated using corn oil, Tween 80, and propylene glycol, followed by solidification onto Neusilin® US2 to obtain solid SNEDDS (S-SNEDDS). Physicochemical properties, including flowability, bulk and tapped density, and residual moisture content, were *evaluated* to assess processability and stability. *In vivo* biodistribution and hepatic targeting were investigated in mice using IVIS Spectrum fluorescence imaging (700-900 nm) after oral administration of three formulations: BSRE + corn oil + DiR, blank SNEDDS + DiR, and SNEDDS-BSRE + DiR. Quantitative region-of-interest (ROI) analysis was performed using Living Image® software, and statistical differences were analyzed by one-way ANOVA followed by Tukey's post-hoc test. The S-SNEDDS granules exhibited excellent flow properties (angle of repose = 27°, Carr's index = 13.5%, Hausner's ratio = 1.16) and optimal residual moisture (3.20%), confirming good solid-state stability and reconstitution performance. IVIS imaging revealed formulation-dependent biodistribution profiles: BSRE + corn oil produced diffuse systemic fluorescence, blank SNEDDS + DiR showed weak and transient signals, whereas SNEDDS-BSRE + DiR demonstrated moderate but sustained hepatic fluorescence. Quantitative ROI analysis confirmed significant intergroup differences (p < 0.001). Overall, incorporation of BSRE into the SNEDDS matrix enhanced nanocarrier stability and hepatic localization, highlighting the potential of SNEDDS-BSRE as a bioimaging-guided oral nutraceutical platform for liver-targeted delivery.

## 1. Introduction

Liver diseases constitute a major and growing global health burden arising from exposure to hepatotoxins, excessive alcohol consumption, viral infections, and metabolic disorders such as non-alcoholic fatty liver disease (NAFLD). Chronic liver disorders are responsible for more than two million deaths annually worldwide, highlighting the urgent need for effective preventive and therapeutic interventions. As the primary organ responsible for metabolism and detoxification, the liver requires sufficient and sustained delivery of hepatoprotective agents to achieve therapeutic concentrations while minimizing premature degradation and systemic clearance. However, many natural hepatoprotective phytoconstituents, particularly flavonoids and anthocyanins, exhibit poor aqueous solubility, limited stability in the gastrointestinal tract, and extensive first-pass metabolism, which collectively restrict their oral bioavailability and clinical translation 1. To address these challenges, lipid-based nanocarrier systems, especially Self-Emulsifying Drug Delivery Systems (SEDDS) and their nanoscale counterparts (SNEDDS), have gained increasing attention. Upon exposure to gastrointestinal fluids, SNEDDS spontaneously form fine oil-in-water nanoemulsions that markedly enhance solubilization and dissolution of poorly water-soluble compounds 2. In addition, these systems promote intestinal permeability and lymphatic uptake, thereby partially bypassing presystemic hepatic metabolism and improving oral bioavailability 3. Beyond pharmacokinetic enhancement, SNEDDS protect labile bioactives from enzymatic degradation, prolong the absorption window, and enable controlled biodistribution, making them particularly suitable for complex natural products with synergistic pharmacological profiles 4.

Black Sticky Rice Extract (BSRE; *Oryza sativa* var. *glutinosa* Blanco) represents a rich source of bioactive phytochemicals, dominated by anthocyanins, particularly cyanidin-3-O-glucoside (Cy3G), along with diverse phenolic and flavonoid compounds 5. These constituents have been widely reported to exert potent antioxidant, anti-inflammatory, and hepatoprotective effects through modulation of oxidative stress and inflammatory pathways, including NF-κB and TNF-α signaling, in experimental models of liver injury 6. Despite its promising biological activity, the therapeutic application of BSRE remains limited due to the inherent instability of anthocyanins. These compounds are highly susceptible to degradation under physiological conditions, including pH variation, enzymatic hydrolysis, and microbial metabolism in the gastrointestinal tract, resulting in poor intestinal absorption and rapid systemic elimination 7. Lipid-based nanoformulation strategies, particularly SNEDDS, offer a rational approach to overcoming these limitations. Upon oral administration, SNEDDS generate nanosized emulsions that enhance the solubilization of lipophilic and semipolar constituents, protect unstable anthocyanins from degradation, and facilitate absorption via intestinal lymphatic pathways 8. Importantly, SNEDDS have been shown to improve both the bioavailability and pharmacokinetic stability of polyphenols and flavonoids compared with conventional extract formulations 9. However, despite extensive reports on bioavailability enhancement, systematic evaluation of the *in vivo* biodistribution of SNEDDS, especially with respect to hepatic localization, remains limited. Therefore, the present study aimed to investigate the *in vivo* biodistribution of DiR-labeled solid Self-Nanoemulsifying Drug Delivery Systems (S-SNEDDS) incorporating Black Sticky Rice Extract using IVIS Spectrum fluorescence imaging. The optimized formulation demonstrated favorable physicochemical characteristics, including droplet size, stability, and reconstitution behavior. Comparative bioimaging and quantitative region-of-interest (ROI) analyses were performed across three formulations: BSRE + corn oil + DiR, blank SNEDDS + DiR, and SNEDDS-BSRE + DiR, to elucidate formulation-dependent biodistribution patterns and hepatic targeting efficiency in live mice.

## 2. Materials and Methods

### 2.1. Materials

Black sticky rice (*Oryza sativa* var. *glutinosa* Blanco) was obtained from Plawad Village, Karawang Timur District, Karawang, West Java, Indonesia. The plant material was taxonomically authenticated at the Herbarium of the School of Life Sciences and Technology (SITH), Institut Teknologi Bandung, Indonesia. The crude extract was prepared by PT. Phytochemindo Reksa (Bogor, West Java, Indonesia) using maceration with 70% ethanol as the extraction solvent. The near-infrared (NIR) fluorescent dye DiR was purchased from TargetMol Chemicals (Boston, MA, USA). Various liquid lipids were obtained commercially. Tween® 20 and Tween® 80 were purchased from Sigma-Aldrich (Madrid, Spain). All other solvents and reagents were of analytical grade and used as received 10.

### 2.2. Animals

All animal experiments were conducted in accordance with international ethical standards for the care and use of laboratory animals. The experimental protocol received approval from the Health Research Ethics Committee, National Research and Innovation Agency (BRIN), Indonesia (Ethics Approval No.: 004/KE.03/SK/01/2025, Ethics Approval on Health Research). Male Balb/c mice (*Mus musculus*), weighing 25-35 g, were used in this study. Animals were maintained under standard laboratory conditions (temperature 22 ± 2 °C, 12 h light/dark cycle) with free access to pellet diet and water ad libitum.

### 2.3. Solidification and Characterization of Solid-SNEDDS (S-SNEDDS)

The SNEDDS formulation containing Black Sticky Rice Extract (BSRE) was transformed into a Solid Self-Nanoemulsifying Drug Delivery System (S-SNEDDS) through an adsorption technique. Neusilin® US2, a highly porous magnesium aluminometasilicate, was employed as the solid carrier owing to its extensive surface area and high oil adsorption capacity. The SNEDDS preconcentrate was gradually incorporated into Neusilin® US2 under continuous mixing until a free-flowing powder was obtained, which was subsequently sieved through a 100-mesh screen to achieve uniform particle size distribution. The physicochemical characteristics of the S-SNEDDS were evaluated for flowability and compressibility by determining bulk density, tapped density, Carr's Index, and Hausner's Ratio in accordance with pharmacopeial procedures (USP <616>). The drug content was determined by dissolving an accurately weighed portion of S-SNEDDS in a suitable solvent, followed by chromatographic quantification at the characteristic absorption wavelength of the active constituent. All analyses were conducted in triplicate, and results were reported as mean ± standard deviation (SD).

### 2.4. Quantitative Organ Biodistribution Analysis of DiR-Labeled Nanocarriers Using *In Vivo* Imaging System (IVIS)

The biodistribution of DiR-labeled nanocarriers was assessed using an *in vivo* imaging system (IVIS Spectrum, PerkinElmer, USA) to visualize and quantify fluorescence signals in live mice. Balb/c mice (*Mus musculus*) were randomly divided into three groups (n = 3 per group) and administered the following formulations via oral gavage: (1) BSRE + corn oil + DiR, (2) blank SNEDDS + DiR, and (3) SNEDDS-BSRE + DiR. Each formulation contained an equivalent DiR dose of 3 mg/kg body weight. At predetermined time intervals (0.5, 1, 2, 3, 4, and 6 h post-administration), animals were anesthetized with isoflurane and positioned in the IVIS chamber in both ventral and dorsal orientations. Fluorescence emission was captured using excitation and emission filters set at 748 nm and 780 nm, respectively. All acquisition parameters were maintained constant throughout the study to ensure comparability of fluorescence intensity among formulations and time points. Regions of interest (ROIs) were manually delineated over the liver, intestine, and abdominal cavity using Living Image® software (version 4.5; PerkinElmer, USA). Quantitative fluorescence intensity within each ROI was expressed as radiant efficiency [(p/s/cm²/sr)/(µW/cm²)]. Background fluorescence (BKG1) was subtracted from each image to correct for non-specific signal. For comparative analysis, mean fluorescence values for each ROI were plotted as bar charts to determine the relative hepatic accumulation of DiR among the tested formulations. The BSRE + corn oil + DiR group exhibited the highest total radiant efficiency, indicating diffuse systemic dispersion. In contrast, the SNEDDS-BSRE + DiR formulation demonstrated moderate but localized hepatic fluorescence, suggesting enhanced targeting specificity and stability of DiR within the nanoemulsion matrix.

## 3. Results & Discussion

### 3.1. Flowability Characterization of S-SNEDDS

#### 3.1.1. Moisture Content

**Figure 1:**
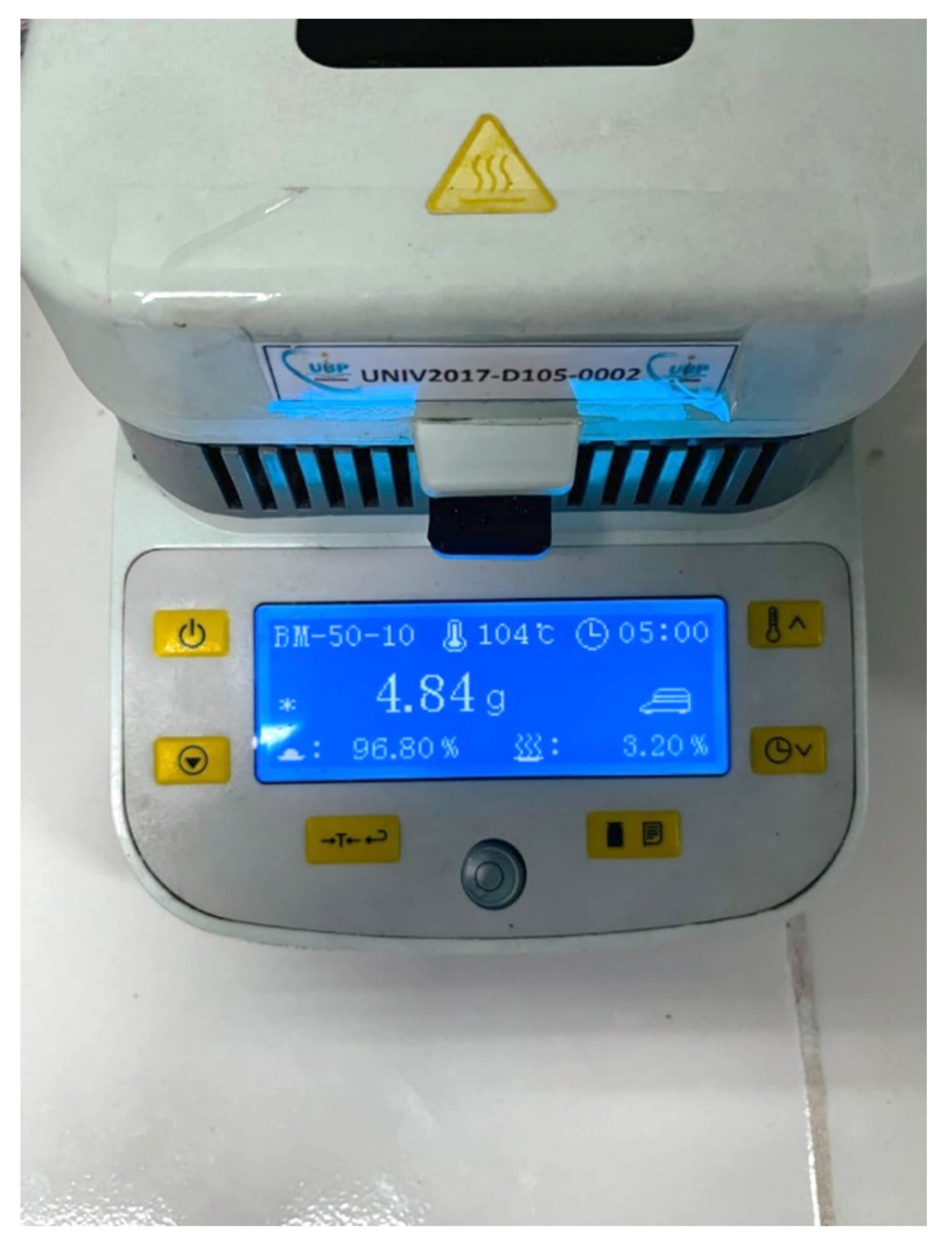
Moisture content of solid S-SNEDDS granules adsorbed onto Neusilin® US2, determined using a halogen moisture analyzer (104 °C, 5 min).

Figure 1 presents the residual moisture content of solidified SNEDDS granules adsorbed onto Neusilin® US2, determined using a halogen moisture analyzer (BM-50-10, Japan). The drying process at 104 °C yielded a residual moisture level of 3.20 %, indicating effective solvent removal and suitability for further solid-state characterization. The residual moisture level of 3.20 % confirms adequate dehydration of the S-SNEDDS granules while preserving formulation integrity. Moisture contents within the range of 2-5 % are generally considered acceptable for lipid-based solid dispersions, as excessive water may induce aggregation, hydrolytic degradation, or compromised flowability 11. The optimized drying condition ensured sufficient water removal without exposing thermolabile lipid and polyphenolic constituents to excessive thermal stress. Maintaining low residual moisture is essential for ensuring the physical stability, flow properties, and reconstitution performance of solidified SNEDDS formulations. Moderate residual moisture also enhances compactability and reconstitution efficiency, ensuring the formation of fine and reproducible nanoemulsion droplets upon dilution. The observed moisture level is consistent with efficient adsorption of SNEDDS into the porous matrix of Neusilin® US2, facilitating stable solidification without inducing capillary liquid bridging or particle agglomeration. This behavior is consistent with the high porosity and oil adsorption capacity of Neusilin® US2, which enables uniform distribution of lipid droplets within its internal structure 12.

#### 3.1.2. Flowability and Angle of Repose

**Figure 2:**
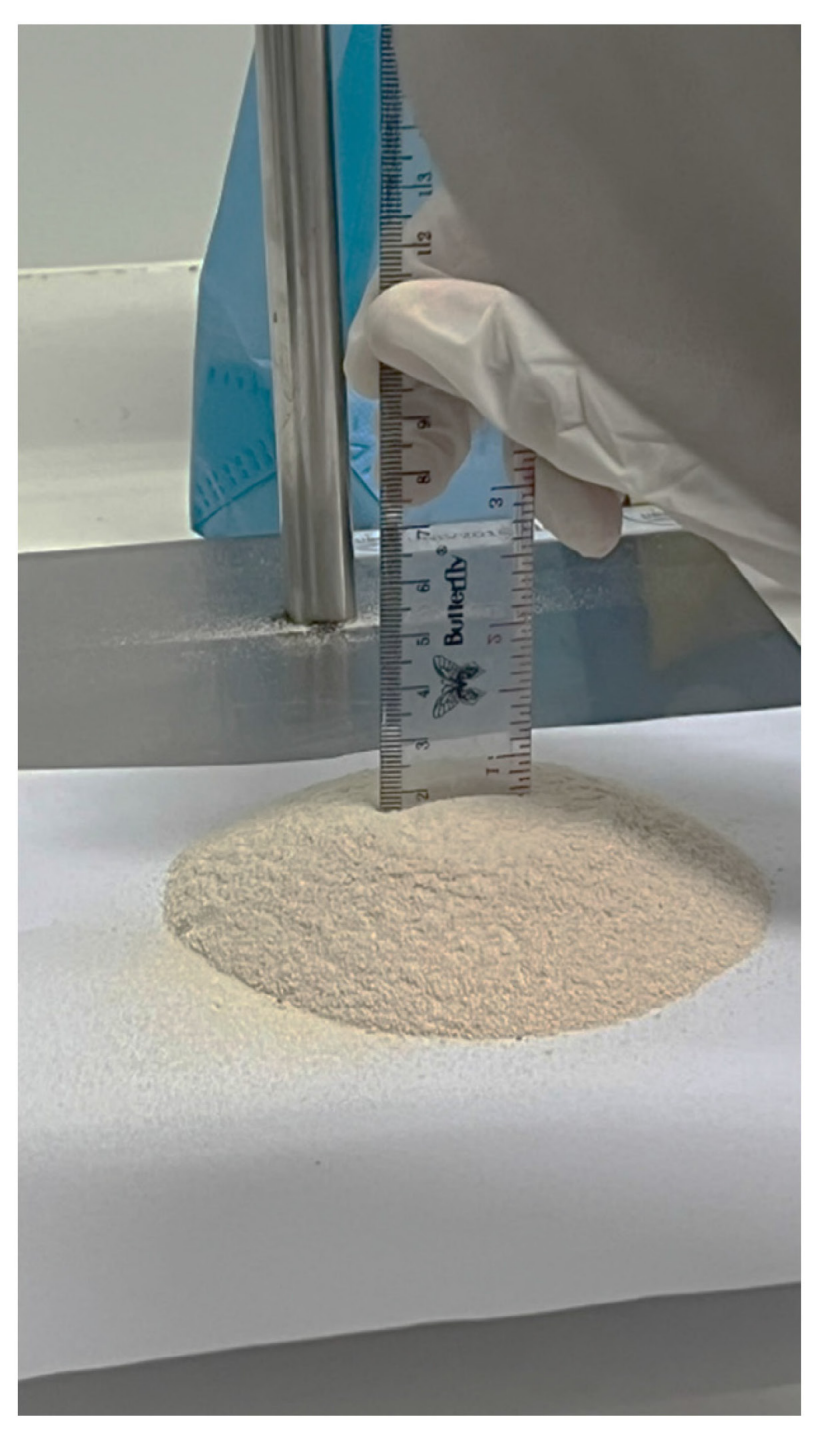
assessment of solid S-SNEDDS granules using the fixed-funnel method for angle of repose determination.

Figure 2 illustrates the flowability assessment of the solidified SNEDDS granules using a fixed-funnel method. The measured angle of repose ranged between 25° and 30°, corresponding to good-to-excellent flow behavior according to pharmacopeial criteria (USP <1174>). The favorable flow properties indicate low interparticulate friction and minimal cohesiveness, which are critical for ensuring uniform die filling and capsule weight consistency. The transformation of liquid SNEDDS into free-flowing granules was achieved through adsorption onto Neusilin® US2, effectively mitigating the inherent stickiness of lipid-based systems. The excellent flow behavior is largely due to the porous and irregular surface morphology of Neusilin® US2, which allows uniform embedding of SNEDDS droplets and minimizes adhesive contact between particles. This behavior is consistent with the high porosity and adsorption capacity of Neusilin® US2, which has been widely reported to enhance flowability and handling characteristics of solidified lipid formulations. Adequate moisture control further contributed to the suppression of liquid bridge formation, maintaining powder flow stability 13,14.

#### 3.1.3. Bulk and Tapped Density, Carr's Index, and Hausner's Ratio

**Figure 3:**
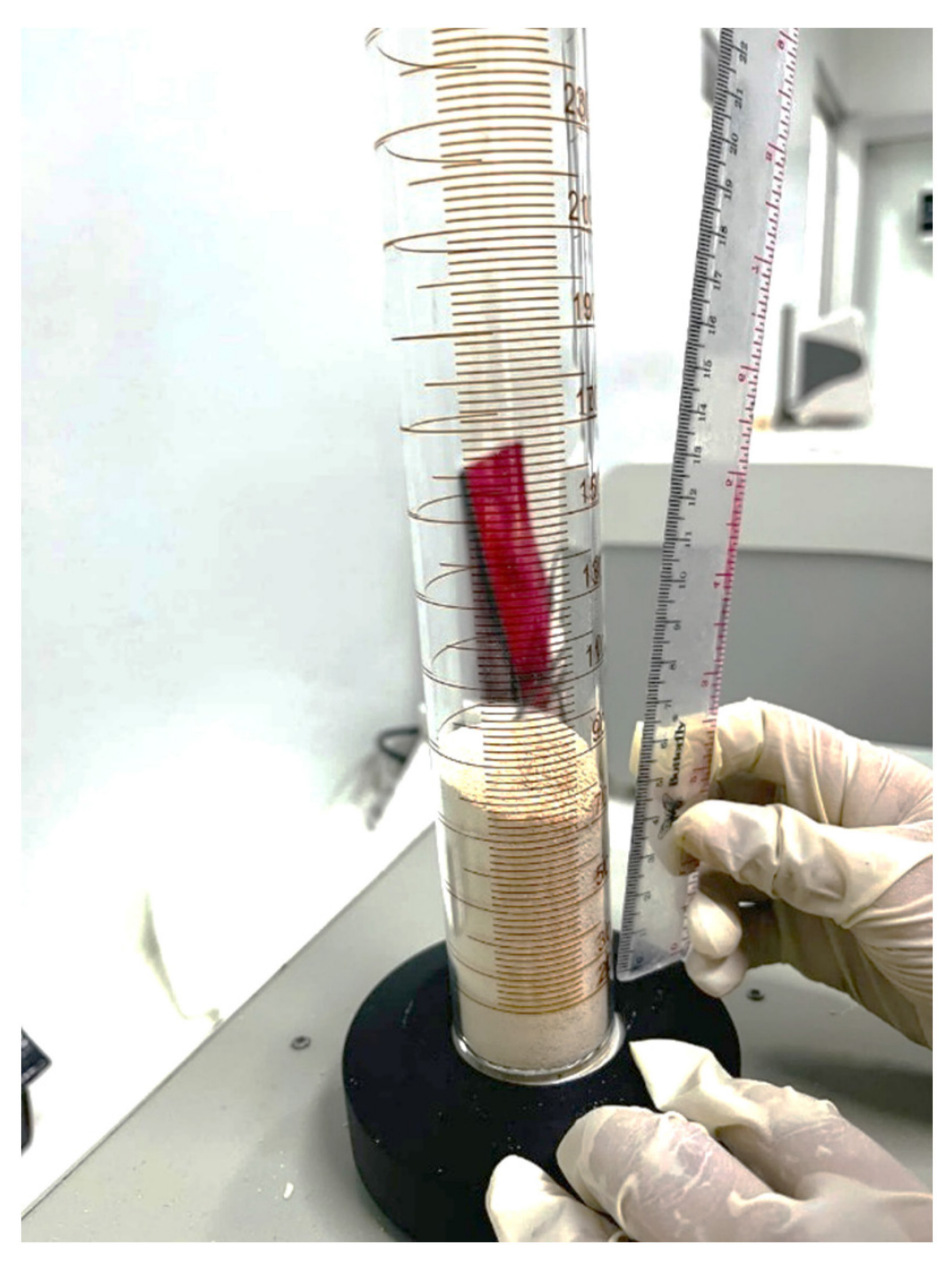
Determination of bulk and tapped density of S-SNEDDS granules for flowability and compressibility evaluation.

Figure 3 depicts the determination of bulk and tapped density of S-SNEDDS granules using a graduated cylinder. The bulk density was approximately 0.45 g/cm³, increasing to 0.52 g/cm³ after tapping. These values yielded a Carr's Index of 13.5 % and a Hausner's Ratio of 1.16, indicative of excellent flowability and low interparticle cohesion (USP <616>). The modest increase in density upon tapping reflects minor particle rearrangement without significant compaction, consistent with the presence of low-density, highly porous carrier particles. The favorable CI and HR values confirm efficient powder packing behavior and mechanical robustness, supporting downstream manufacturing processes such as capsule filling. The outstanding flow behavior of the S-SNEDDS can be attributed to the adsorptive solidification mechanism in which liquid SNEDDS droplets are uniformly distributed within the mesoporous framework of Neusilin® US2. This behavior is consistent with the high porosity and adsorption capacity of Neusilin® US2, which promotes uniform lipid distribution while minimizing electrostatic interactions and interparticle bridging. Similar improvements in flow and packing efficiency have been reported for Neusilin-based S-SNEDDS systems, demonstrating their suitability for scalable solid oral dosage form production 15.

### 3.2. Quantitative Organ Biodistribution Analysis of DiR-Labeled Nanocarriers Using *In Vivo* Imaging System (IVIS)

**Figure 5:**
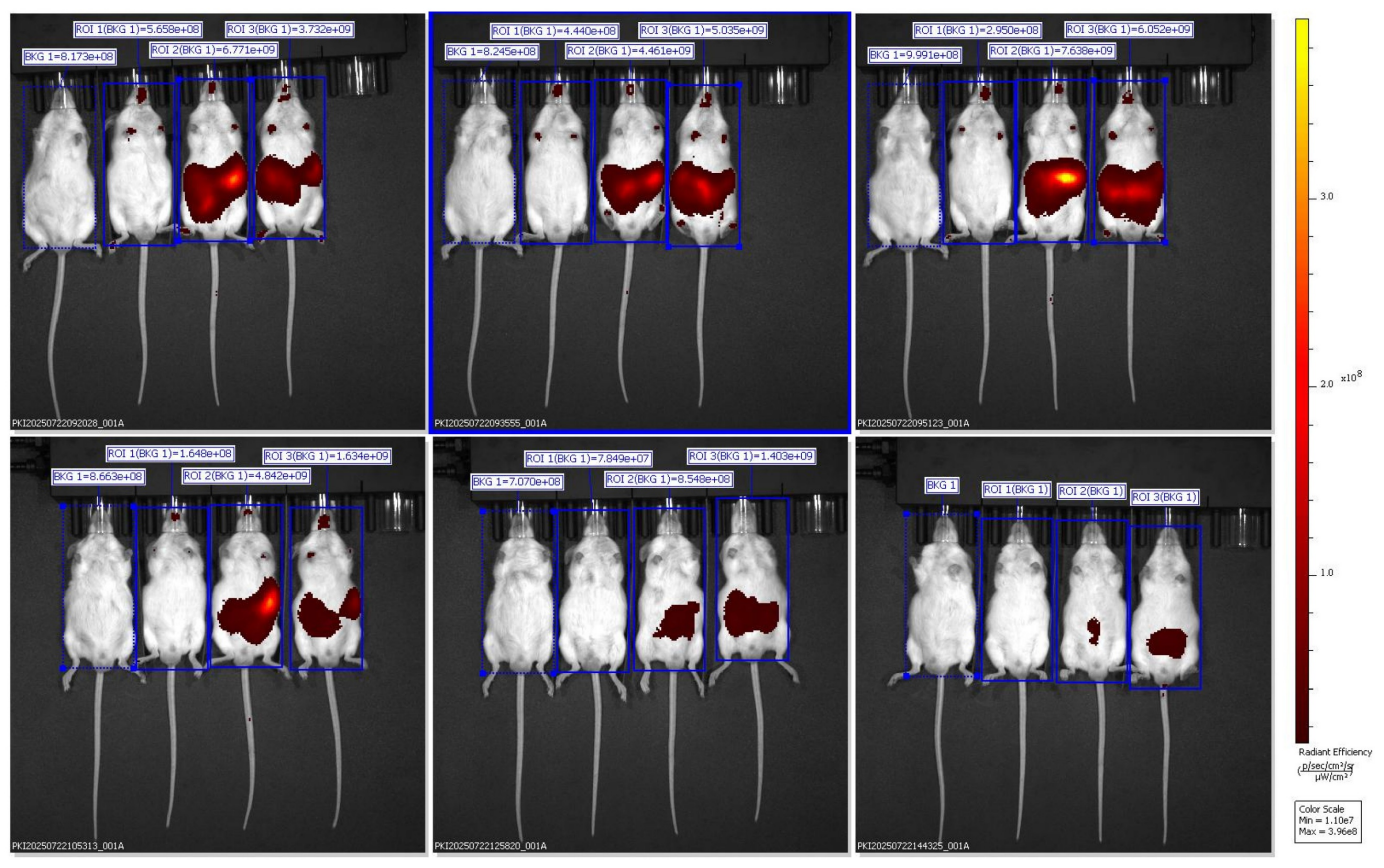
Representative ventral IVIS images showing DiR distribution following administration of SNEDDS + DiR formulation.

**Figure 6:**
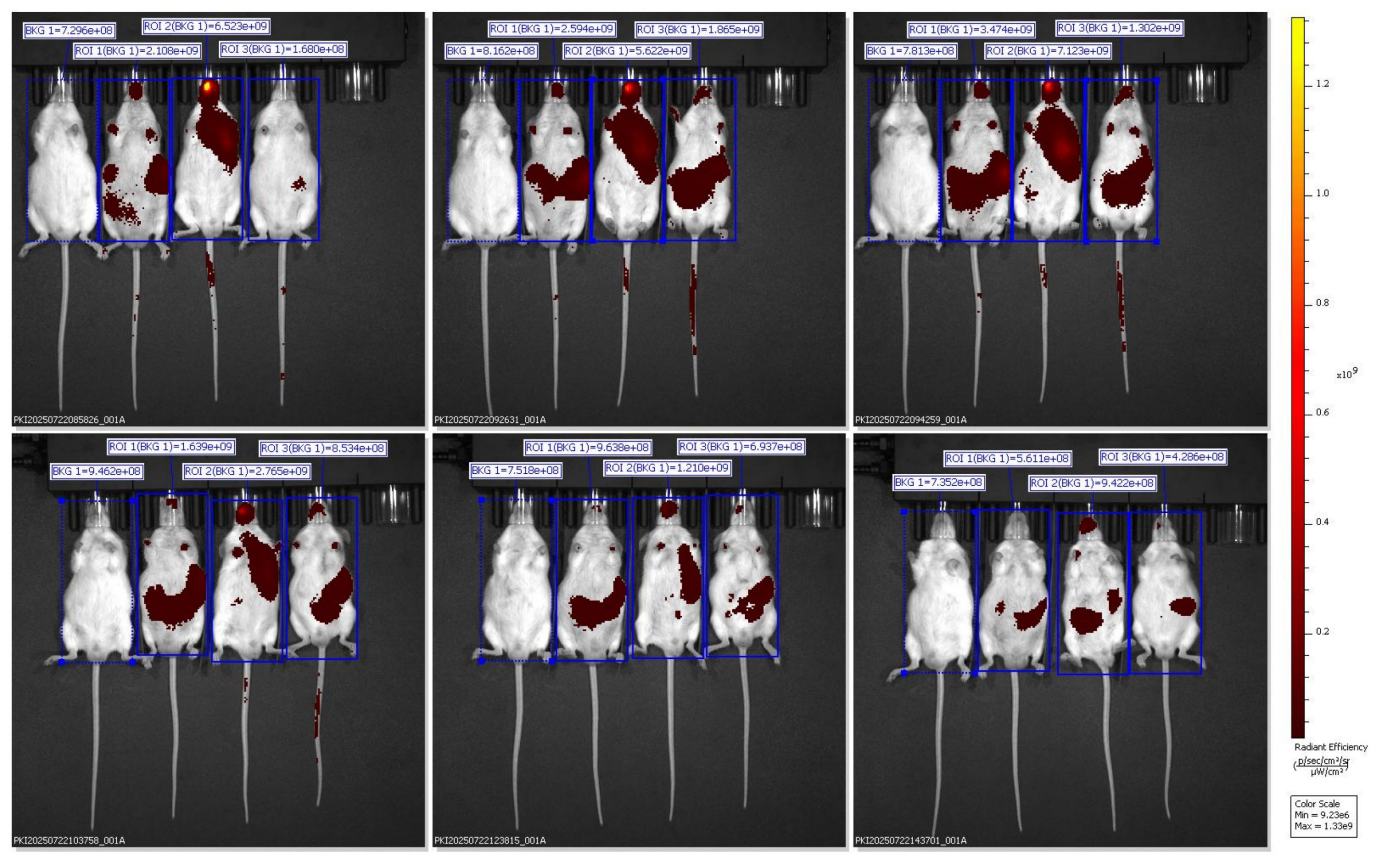
Ventral IVIS imaging of mice receiving SNEDDS-BSRE + DiR formulation showing preferential hepatic localization.

*In vivo* fluorescence images were captured using the IVIS Spectrum Imaging System in the ventral (supine) position at multiple time points (30 min to 6 h) after oral administration of Black Sticky Rice Extract (BSRE) dissolved in corn oil containing DiR dye. The pseudocolor scale (right) represents fluorescence intensity expressed as radiant efficiency ((p/s/cm²/sr)/(µW/cm²)). Intense and widespread signals were observed across the abdominal and thoracic regions, suggesting systemic dispersion of the lipophilic tracer rather than specific hepatic accumulation. The *in vivo* fluorescence imaging of mice administered with BSRE + Corn Oil + DiR revealed a broad and intense fluorescent signal distributed across the abdominal and thoracic regions. The emission intensity reached its maximum within the first 1-2 h after oral administration and gradually declined by 6 h. This pattern indicates that the DiR tracer, a highly lipophilic near-infrared (NIR) fluorophore, underwent rapid systemic dispersion rather than organ-specific targeting. The observed fluorescence throughout the body corresponds to the nonspecific absorption and circulation of DiR encapsulated in the lipidic corn oil medium 16,17. Such wide biodistribution is consistent with the physicochemical characteristics of DiR, which preferentially partitions into lipid-rich environments and integrates within plasma membranes. When administered with simple lipid vehicles (such as corn oil), DiR primarily follows chylomicron-mediated transport through intestinal lymphatic uptake, subsequently entering systemic circulation via thoracic lymphatic drainage. This explains the strong fluorescence observed across the thoracic cavity and upper abdomen in the ventral imaging view. However, the lack of specific hepatic localization suggests that the lipid vehicle alone (corn oil) does not effectively direct DiR or BSRE components toward targeted hepatic accumulation. Instead, the formulation likely promotes nonspecific dispersion into adipose tissues and systemic lipid compartments, consistent with previous studies demonstrating that simple triglyceride-based vehicles lack the surfactant-mediated control of droplet size and distribution necessary for targeted biodistribution 18. From a pharmacokinetic standpoint, this result supports the hypothesis that BSRE in corn oil behaves as a conventional lipid dispersion, showing good intestinal absorption but limited organ specificity. The high radiant efficiency in the ventral projection primarily reflects DiR's lipophilicity and its incorporation into circulating lipoproteins, not active tissue targeting. This contrasts with the more localized hepatic accumulation observed in SNEDDS and SNEDDS-BSRE formulations (Figures 5 and 6), which exhibit more controlled dispersion profiles 19,20. The findings therefore highlight that while corn oil enhances solubilization of lipophilic compounds such as BSRE and DiR, it does not provide the nanometric emulsification or mucoadhesive properties required for hepatic targeting or sustained residence. These observations align with the established understanding that the transformation of lipid vehicles into self-emulsifying or nanoemulsifying systems is necessary to achieve predictable biodistribution and enhanced bioavailability 21.

Lipophilic carbocyanine dyes such as DiR may partially dissociate from nanocarrier interfaces in biological media and redistribute to plasma lipoproteins and cellular membranes, leading to attenuated focal fluorescence signals and potentially misleading biodistribution profiles when no stabilizing bioactive cargo is present. This phenomenon is further compounded by partial dye aggregation and aggregation-caused quenching (ACQ), which can reduce detectable fluorescence following DiR release from unstable lipid interfaces. Previous studies have demonstrated that DiR readily diffuses out of lipid-based nanocarriers, emphasizing that fluorescence intensity may reflect tracer kinetics rather than true nanocarrier localization. In line with these observations, the weak and transient fluorescence detected in the blank SNEDDS + DiR group likely reflects limited interfacial stability and rapid systemic clearance, rather than inadequate imaging sensitivity. In contrast, incorporation of BSRE appears to enhance interfacial stabilization and prolong nanocarrier residence, resulting in more localized hepatic fluorescence. Consistent with recommended standards for quantitative optical imaging, future biodistribution studies should incorporate free-dye controls and *ex vivo* organ imaging to reliably distinguish nanocarrier-mediated targeting from dye redistribution effects 22.

Ventral IVIS imaging demonstrated that oral administration of SNEDDS-BSRE + DiR resulted in a distinct and localized fluorescence signal predominantly in the upper abdominal region corresponding anatomically to the liver. In contrast to the widespread systemic dispersion observed with BSRE + corn oil and the weak, transient signal from blank SNEDDS, the BSRE-loaded SNEDDS exhibited moderate yet sustained hepatic fluorescence up to 6 h post-administration. This biodistribution pattern suggests improved tracer retention and reduced nonspecific redistribution when DiR is incorporated within a stabilized nanoemulsion matrix. The presence of polyphenolic constituents in BSRE, including cyanidin-3-O-glucoside and quercetin, likely contributes to interfacial stabilization of the lipid droplets through hydrogen bonding and π-π interactions, thereby limiting dye leakage and aggregation-caused quenching effects commonly reported for carbocyanine probes such as DiR 23,24. Moreover, the fine oil droplets generated by SNEDDS favor intestinal lymphatic transport and subsequent hepatic exposure via chylomicron-mediated uptake, supporting the observed liver-preferring distribution 25. Collectively, these findings indicate that BSRE incorporation enhances nanoemulsion stability and promotes more localized hepatic biodistribution while minimizing systemic signal dispersion, underscoring the suitability of SNEDDS-BSRE as an oral platform for liver-targeted delivery.

The dorsal IVIS images further validate the preferential hepatic accumulation of the SNEDDS-BSRE + DiR formulation. Localized fluorescence hotspots beneath the rib cage region confirm liver-specific signal confinement rather than gastrointestinal retention or diffuse systemic dispersion 26. In contrast to BSRE + corn oil and blank SNEDDS, which exhibited widespread or rapidly diminishing signals, the SNEDDS-BSRE system produced moderate yet sustained hepatic fluorescence up to 4 h post-administration. This behavior suggests improved nanocarrier integrity and tracer retention, likely mediated by stabilizing interactions between BSRE polyphenols (e.g., cyanidin-3-O-glucoside and quercetin) and the lipid-surfactant interface. Such interactions reduce dye leakage and delay systemic redistribution, consistent with reports on enhanced interfacial stability in polyphenol-loaded lipid nanocarriers 27-29. Additionally, the nanoscale droplet size of SNEDDS facilitates intestinal lymphatic uptake and subsequent hepatic exposure via chylomicron-mediated transport, contributing to the observed liver confinement 30,31. Collectively, the dorsal imaging data strengthen the conclusion that BSRE incorporation is essential for achieving controlled hepatic biodistribution while minimizing nonspecific systemic exposure, supporting the suitability of SNEDDS-BSRE as an oral delivery platform for hepatoprotective agents.

Quantitative ROI analysis (Table 1) revealed formulation-dependent differences in fluorescence biodistribution and tracer retention. One-way ANOVA demonstrated a significant difference among groups (F(2,6) = 18.47, p < 0.001). Post-hoc Tukey's analysis showed that the BSRE + Corn Oil + DiR group exhibited the highest radiant efficiency, consistent with widespread systemic fluorescence mediated by nonspecific lipid transport. In contrast, the blank SNEDDS + DiR formulation produced the lowest and most transient fluorescence signals, reflecting rapid systemic clearance and limited tissue retention, likely due to dye leakage from unstable nanocarrier interfaces. Notably, the SNEDDS-BSRE + DiR formulation displayed a moderate but sustained fluorescence intensity predominantly localized in the hepatic region. This controlled biodistribution suggests enhanced nanocarrier stability and reduced tracer dissociation, attributed to the presence of BSRE polyphenols, including cyanidin-3-O-glucoside and quercetin, which can stabilize the lipid-surfactant interface via hydrogen bonding and π-π interactions. In addition, the antioxidant properties of BSRE may suppress lipid peroxidation, thereby preserving nanoemulsion integrity during gastrointestinal transit and systemic circulation 32,33. The hepatotropic distribution observed for SNEDDS-BSRE is consistent with lymphatic absorption of lipid-based nanocarriers followed by preferential hepatic uptake via reticuloendothelial system pathways 34. Collectively, these quantitative findings corroborate the IVIS imaging results and confirm that incorporation of BSRE into the SNEDDS matrix enhances tracer stability, promotes hepatic localization, and minimizes nonspecific systemic dispersion. This balanced biodistribution profile supports the potential of SNEDDS-BSRE as an effective oral delivery platform for liver-targeted nutraceutical or therapeutic applications.

**Figure 8:**
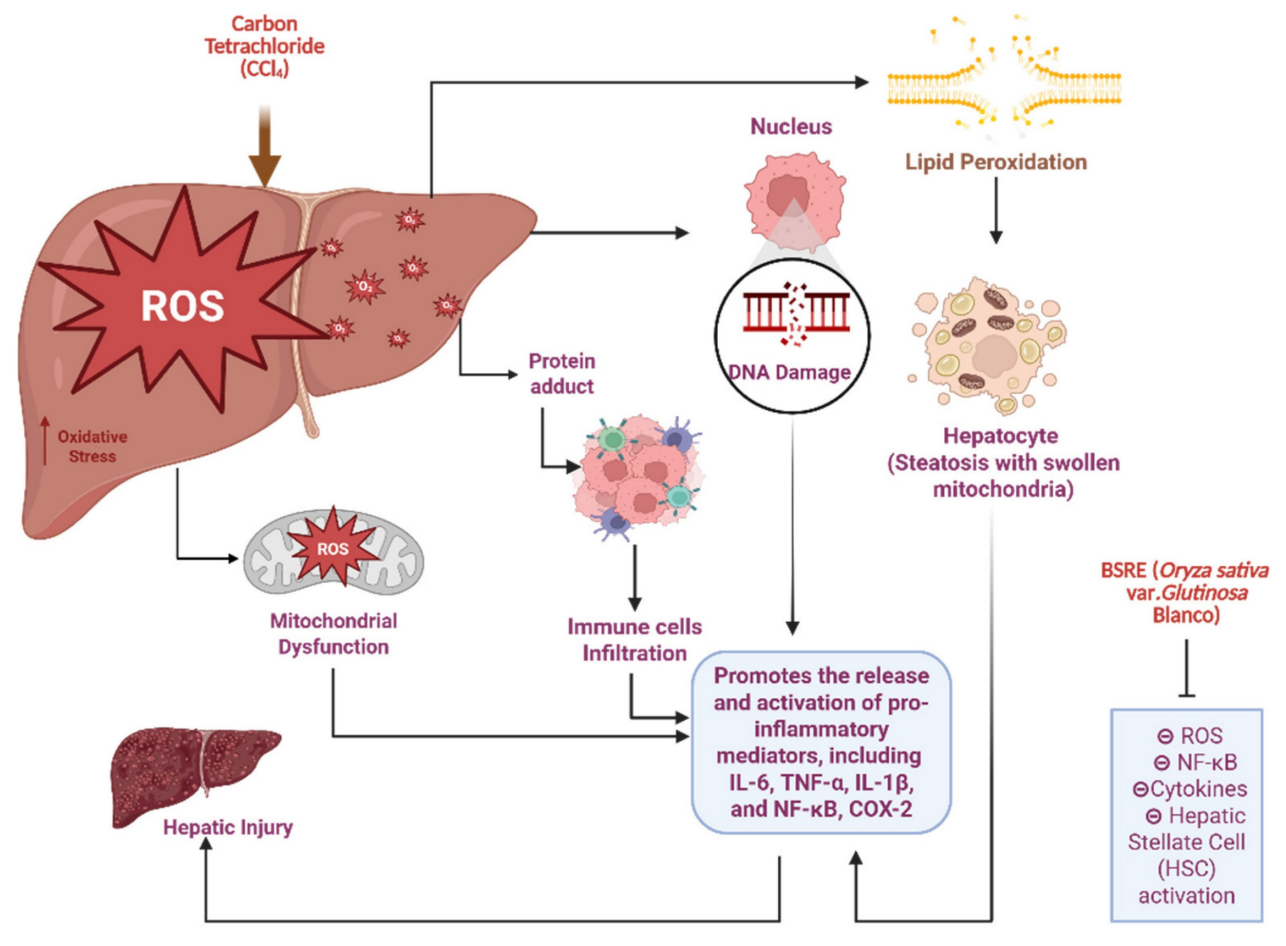
Proposed mechanism of CCl₄-induced hepatotoxicity and hepatoprotective action of Black Sticky Rice Extract (BSRE). (Biorender.com).

Figure 8 is presented to provide biological context for the hepatoprotective relevance of BSRE and does not represent an experimental outcome of the present biodistribution study. Hepatic biotransformation of CCl₄ via cytochrome P450 2E1 generates highly reactive trichloromethyl radicals, initiating lipid peroxidation, mitochondrial dysfunction, and oxidative DNA damage. These events activate NF-κB-mediated inflammatory cascades, upregulating pro-inflammatory cytokines (TNF-α, IL-1β, IL-6) and COX-2, ultimately leading to hepatocyte necrosis and progression toward fibrosis 35. This pathophysiological sequence is well established in experimental models of CCl₄-induced liver injury and provides a relevant framework for evaluating hepatoprotective interventions. BSRE administration attenuates this toxic cascade through the combined antioxidant and anti-inflammatory activities of its anthocyanin and flavonoid rich matrix, predominantly cyanidin-3-O-glucoside and quercetin. These polyphenols scavenge reactive oxygen species, suppress lipid peroxidation, preserve mitochondrial integrity, and downregulate NF-κB signaling while promoting Nrf2-dependent antioxidant defenses 36. The preferential hepatic localization observed for SNEDDS-BSRE in the present biodistribution study supports this mechanism by enhancing local exposure of BSRE bioactives to hepatically perfused tissues. Together, these findings suggest that BSRE-loaded SNEDDS function as a dual-acting system, combining stabilized nanocarrier delivery with intrinsic hepatoprotective bioactivity, thereby mitigating oxidative stress-driven liver injury 37,38. When interpreting the sustained liver-weighted signal observed for SNEDDS-BSRE likely reflects improved interfacial stability and lymphatic transport rather than tracer kinetics alone. Future studies incorporating free-dye controls and *ex vivo* organ quantification will further substantiate organ-specific targeting.

## Conclusion

This study successfully developed a DiR-labeled Self-Nanoemulsifying Drug Delivery System (SNEDDS) incorporating Black Sticky Rice Extract (BSRE) and demonstrated its effective solidification using Neusilin® US2 as a porous carrier, yielding a physically stable, free-flowing powder with excellent reconstitution performance. *In vivo* fluorescence imaging revealed distinct formulation-dependent biodistribution profiles: BSRE dispersed in corn oil produced diffuse systemic fluorescence, blank SNEDDS exhibited rapid tracer clearance, whereas SNEDDS-BSRE achieved sustained and localized hepatic accumulation with moderate radiant efficiency. The enhanced hepatic confinement of SNEDDS-BSRE is attributed to the synergistic role of BSRE's polyphenolic constituents, particularly anthocyanins and quercetin, which stabilize lipid-surfactant interfaces through hydrogen bonding and π-π stacking interactions, thereby improving nanoemulsion integrity and minimizing dye leakage. Collectively, these findings demonstrate that BSRE-loaded SNEDDS not only improves physicochemical stability and formulation processability, but also enhances hepatic targeting and tracer retention. This platform therefore holds strong potential as a bioimaging-guided, liver-directed nutraceutical delivery system. Future studies involving pharmacokinetic evaluation, *ex vivo* organ fluorescence quantification, and mechanistic validation of Nrf2/NF-κB signaling pathways are warranted to further support its translational applicability for hepatoprotective therapy.

## Figures and Tables

**Figure 4:**
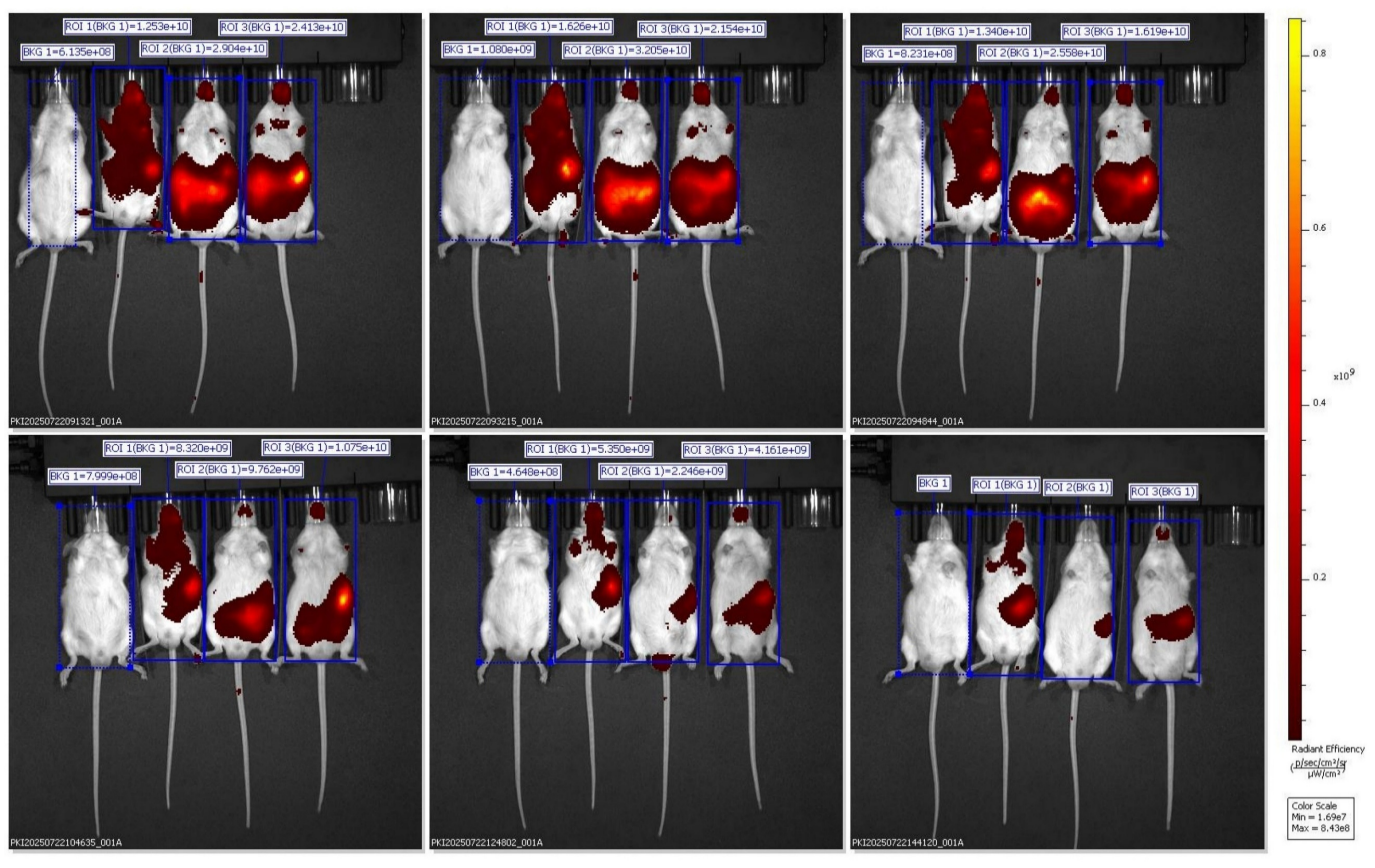
Representative ventral IVIS images showing DiR biodistribution in mice administered with BSRE + corn oil formulation.

**Figure 7:**
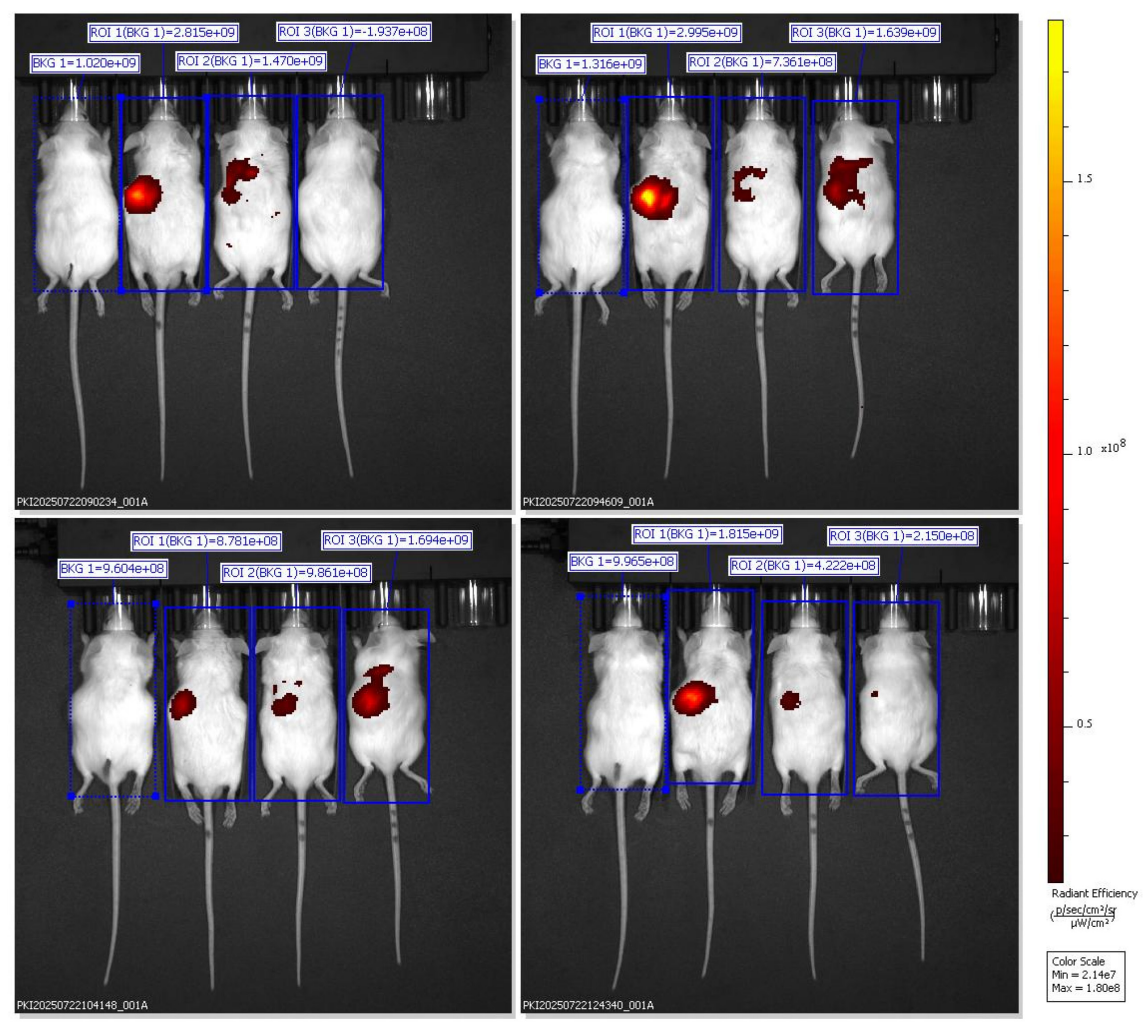
Dorsal IVIS imaging confirms preferential hepatic accumulation of SNEDDS-BSRE + DiR.

**Table 1:** Quantitative ROI analysis of fluorescence biodistribution after oral administration of DiR-labeled formulations. Values are expressed as mean ± SD of radiant efficiency (p/s/cm²/sr)/(µW/cm²), n = 3 mice per group.

Group	Mean ROI (×10⁹)	Fluorescence trend	Biodistribution pattern
BSRE + Corn Oil + DiR	16.2 ± 7.8	High and diffuse	Systemic (non-specific)
SNEDDS + DiR	3.4 ± 2.6	Low and transient	Rapid clearance, minimal retention
SNEDDS-BSRE + DiR	6.3 ± 2.1	Moderate and sustained	Hepatic localization, improved stability
